# Activation of the A2B adenosine receptor in B16 melanomas induces CXCL12 expression in FAP-positive tumor stromal cells, enhancing tumor progression

**DOI:** 10.18632/oncotarget.11729

**Published:** 2016-08-31

**Authors:** Claudia Sorrentino, Lucio Miele, Amalia Porta, Aldo Pinto, Silvana Morello

**Affiliations:** ^1^ Department of Pharmacy, University of Salerno, Fisciano, SA, Italy; ^2^ PhD Program in Drug Discovery and Development, Department of Pharmacy, University of Salerno, Fisciano, SA, Italy; ^3^ Department of Genetics, Louisiana State University Health Sciences Center and Stanley S. Scott Cancer Center, New Orleans, LA, USA

**Keywords:** A2BR, tumor, fibroblasts, CXCL12, mouse melanoma model

## Abstract

The A2B receptor (A2BR) can mediate adenosine-induced tumor proliferation, immunosuppression and angiogenesis. Targeting the A2BR has proved to be therapeutically effective in some murine tumor models, but the mechanisms of these effects are still incompletely understood. Here, we report that pharmacologic inhibition of A2BR with PSB1115, which inhibits tumor growth, decreased the number of fibroblast activation protein (FAP)-expressing cells in tumors in a mouse model of melanoma. This effect was associated with reduced expression of fibroblast growth factor (FGF)-2. Treatment of melanoma-associated fibroblasts with the A2BR agonist Bay60-6583 enhanced CXCL12 and FGF2 expression. This effect was abrogated by PSB1115. The A2AR agonist CGS21680 did not induce CXCL12 or FGF2 expression in tumor associated fibroblasts. Similar results were obtained under hypoxic conditions in skin-derived fibroblasts, which responded to Bay60-6583 in an A2BR-dependent manner, by stimulating pERK1/2. FGF2 produced by Bay60-6583-treated fibroblasts directly enhanced the proliferation of melanoma cells. This effect could be reversed by PSB1115 or an anti-FGF2 antibody. Interestingly, melanoma growth in mice receiving Bay60-6583 was attenuated by inhibition of the CXCL12/CXCR4 pathway with AMD3100. CXCL12 and its receptor CXCR4 are involved in angiogenesis and immune-suppression. Treatment of mice with AMD3100 reduced the number of CD31+ cells induced by Bay60-6583. Conversely, CXCR4 blockade did not affect the accumulation of tumor-infiltrating MDSCs or Tregs. Together, our data reveal an important role for A2BR in stimulating FGF2 and CXCL12 expression in melanoma-associated fibroblasts. These factors contribute to create a tumor-promoting microenvironment. Our findings support the therapeutic potential of PSB1115 for melanoma.

## INTRODUCTION

In the tumor microenvironment stromal cells, including endothelial cells, fibroblasts and several types of immune and inflammatory cells, communicate with neoplastic cells through multiple soluble factors. Stroma-derived growth factors, cytokines and chemokines can directly induce the proliferation and migration of neoplastic cells and support angiogenesis. At the same time, they create a chronic inflammatory milieu that attracts lymphoid and myeloid cells with immunosuppressive features, allowing tumor cells to escape the immune response. Furthermore, tumor stromal cells such as cancer-associated fibroblasts can also induce resistance to chemotherapy and/or targeted therapy, in a soluble factor- and cell adhesion-dependent manner [reviewed in Refs 1 and 2]. Therefore, thoroughly understanding the mechanisms that regulate the interplay between stromal cells and neoplastic cells is of great therapeutic relevance.

Adenosine is a tumor-promoting factor generated from the degradation of ATP, released by inflammatory and/or dying cells in tissue [3]. Extracellular concentrations of adenosine increase in hypoxic tumor tissues because of the up-regulation of adenosine-generating enzyme CD73, expressed on tumor cells and/or host cells [3, 4]. Within the tumor microenvironment, adenosine can affect the behavior of cells including tumor stromal cells and tumor-infiltrating immune cells, protecting tumors from T-cell responses [5]. Among the adenosine receptor subtypes, A2A is the most thoroughly characterized receptor involved in the immunosuppressive effects of adenosine [6]. Inhibitors of CD73 and/or A2A receptor antagonists are referred to as a next generation of immune-checkpoint inhibitors for cancer immunotherapy [6–8].

Over the years, an increasing number of studies have revealed that the A2B receptor (A2BR) plays an important role in tumor-promoting effects of adenosine. A2BR activates multiple downstream pathways, including cAMP and ERK [9]. In contrast to A2AR, A2B is generally active only under pathophysiological conditions when high concentrations of adenosine occur [9], such as in solid tumors. Stimulation of A2BR in tumor cells can promote metastasis [10, 11] while in myeloid cells adenosine binding to A2BR induces immunosuppression [9, 12] and supports angiogenesis together with endothelial cells [13, 14]. Selective A2BR blockade significantly inhibits tumor growth in some murine models [15–20]. However, the mechanisms of these effects remain incompletely understood. Here, we report that A2BR blockade in melanoma-bearing mice reduces the number of fibroblast activation protein (FAP)-positive stromal cells in tumor lesions. We hypothesized that the A2BR may have a role in modulating the activity of stromal fibroblasts. We determined that A2BR stimulation induces the expression of FGF2 and CXCL12 in melanoma-associated fibroblasts. These mediators critically support melanoma cells growth and angiogenesis *in vivo*.

## RESULTS

### The anti-tumor activity of PSB1115 is associated with reduced FAP and FGF2 expression in melanoma tissues

**Figure 1:**
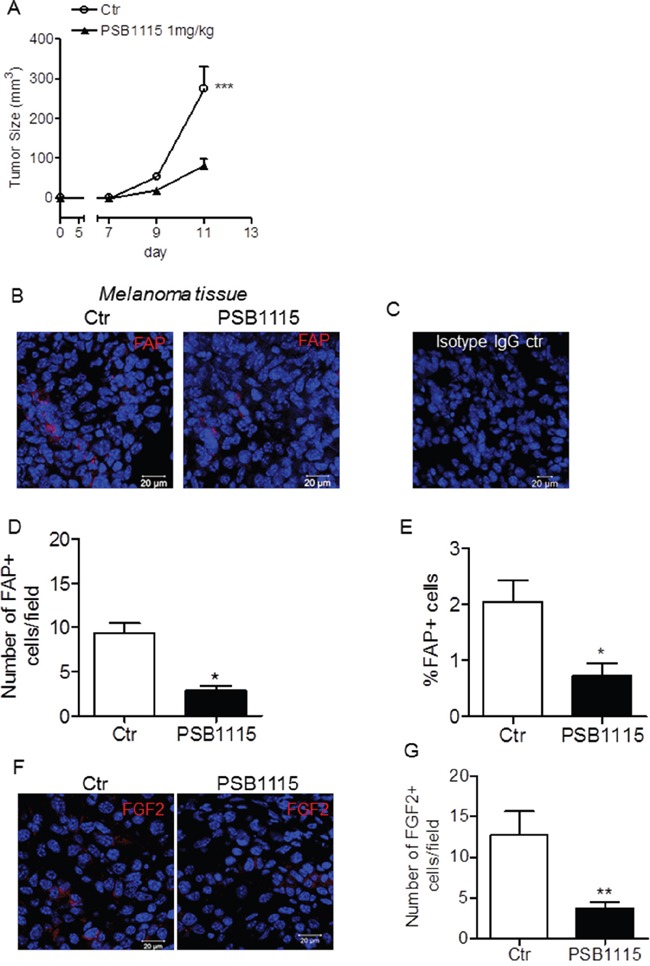
A2BR blockade of *in vivo* reduces fibroblast activation protein (FAP) expression in melanoma tissues **A.** C57Bl6 mice were injected subcutaneously with 2.5 × 10^5^ B16.F10 melanoma cells. On day 6 after tumor cell injection, mice were treated peritumorally with PSB1115 (1 mg/kg) every day for one week. Tumor volume was monitored and calculated as described in Material and Methods. Results are expressed as mean ± SEM. n=11 mice/group. ***p<0.001 as determined by ANOVA. **B.** immunofluorescence images of melanoma sections from C57Bl/6 mice treated with vehicle (control, Ctr) or with PSB1115, a selective A2BR antagonist, stained with an anti-FAP-α specific antibody (red) and counterstained with DAPI (blue). Data are representative of n=6 mice/group. **C.** isotype IgG control did not shown any positive staining. Scale bar, 20 μm. **D.** number of FAP positive cells in control (Ctr) and PSB1115-treated mice. Data are from sections derived from tumors obtained from 6 different mice/group. Two sections were stained for each tumor and positive cells were counted in four to five randomly selected fields per tumor section. **E.** percentage of FAP+ cells analyzed by flow cytometry in melanoma tissues harvested from control mice or PSB1115-treated mice. Data are expressed as mean ± SEM. n=7 mice/group. **F.** representative immunofluorescence images of melanoma sections from control mice or mice treated with PSB1115, stained with an anti-FGF2 specific antibody (red) and counterstained with DAPI (blue). Isotype IgG control did not shown any staining (please refer to panel B). Scale bar, 20 μm. **G.** number of FGF2 positive cells in tumors from control (Ctr) and PSB1115-treated mice counted in four to-five randomly selected fields per tumor section. Data are from sections derived from tumors of 5 mice/group and expressed as mean ± SEM. *, p<0.05 and **, p<0.01 (unpaired *Student*'s t test).

We have previously demonstrated that A2BR blockade inhibits tumor progression in mice (Figure 1A) [17]. To better understand how the A2BR regulates tumor growth within tumor microenvironment, we focused on the activation status of tumor stromal cells. C57Bl6 mice, implanted with B16.F10 melanoma cells, received daily vehicle (phosphate-buffered saline, PBS) or PSB1115, a selective A2BR antagonist (1 mg/kg, p.t.) [19–21]. Melanoma tissues were collected from mice and tumor sections were stained with an antibody anti-fibroblast activation protein α (FAPα), a common marker of activated fibroblasts in tumors [22, 23]. Treatment with PSB1115 led to a reduction in FAP positive cells within tumor tissue (Figure 1B, 1C and 1D). This result was confirmed by flow cytometric analysis of FAP positive cells in melanoma tissues of PSB1115-treated mice versus control (Figure 1E). Notably, analyses performed in tumors of similar sizes, harvested from control mice vs PSB1115-treated mice, showed no correlation between FAP expression and tumor size, suggesting that the treatment of mice with the A2BR inhibitor reduced the number of FAP positive cells in melanoma. Tumor sections of mice treated with PSB1115 also showed reduced expression of basic fibroblast growth factor (FGF)-2 (Figure 1F). Blocking the A2BR with PSB1115 resulted in a significantly lower number of FGF2-positive cells in the tumors compared to control (Figure 1G).

### A2BR stimulation induces CXCL12 expression in FAP-expressing tumor stromal cells

Previous reports show that the A2BR is expressed in fibroblasts [24–26]. Therefore using an anti-A2B antibody (clone N-19) we verified that FAP positive cells in melanoma tissue express A2BR (Supplementary Figure S1).

**Figure 2:**
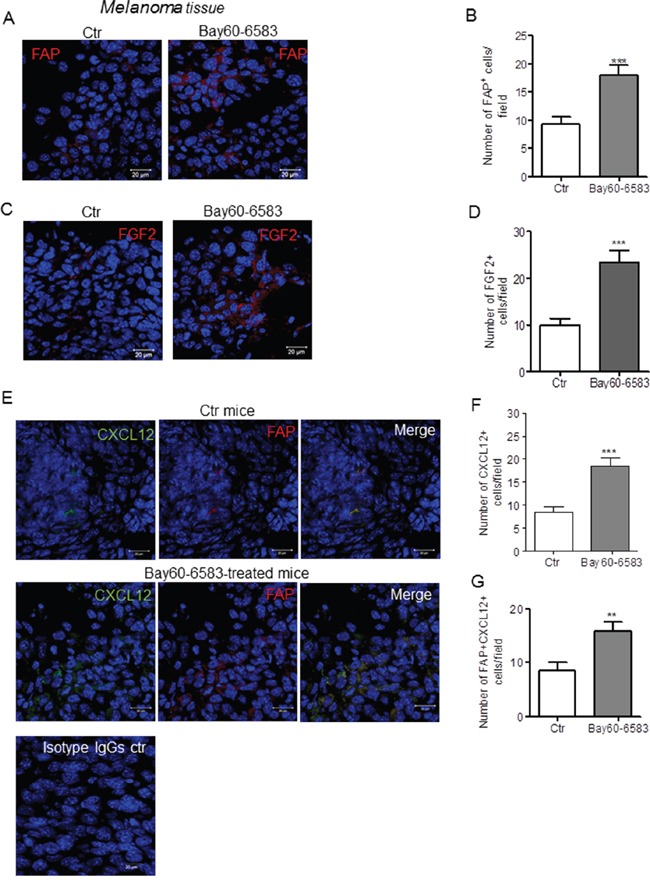
A2BR stimulation enhances the number of FAP positive cells that express CXCL12 and FGF2 in melanoma tissue **A.** representative immunofluorescence images of melanoma sections from mice receiving vehicle (control, Ctr) or mice treated with Bay60-6583, a selective A2BR agonist, stained with anti-FAPα antibody (red) and counterstained with DAPI (blue). Scale bar, 20 μm. **B.** number of FAP positive cells in tumors from control (Ctr) and Bay60-6583-treated mice. Data are from sections derived from tumors obtained from 5 different mice/group. Two sections were stained for each tumor and positive cells were counted in four to five randomly selected fields per tumor section. Results are expressed as mean ± SEM. ***, p<0.001 as determined by Student's t-test. **C.** Immunofluorescence images of melanoma sections from control mice or mice treated with Bay60-6583, stained with anti-FGF2 antibody (red) and counterstained with DAPI (blue). Scale bar, 20 μm. **D.** number of FGF2 positive cells in tumors from control (Ctr) and Bay60-6583-treated mice counted as described above. Results are expressed as mean ± SEM. n=5 mice/group. ***, p<0.001 as determined by Student's t-test **E.** representative immunofluorescence images of melanoma sections from control (Ctr) mice or mice treated with Bay60-6583 stained with an anti-CXCL12 antibody (green) and with an anti-FAPα antibody (red) and counterstained with DAPI (blue). The merged image shows the co-localization of CXCL12 and FAP. Immunofluorescence image of melanoma section stained with normal rabbit IgG and mouse IgG is shown in the lower panel. Scale bar, 20 μm. **F.** number of CXCL12 positive cells in tumors from control (Ctr) and Bay60-6583-treated mice. Data are from sections derived from tumors obtained from 6 different mice/group. Two sections were stained for each tumor and positive cells were counted in four to five randomly selected fields per tumor section. Results are expressed as mean ± SEM. ***, p<0.001 as determined by Student's t-test **G.** quantification of CXCL12 positive FAP positive cells in tumors from control (Ctr) and Bay60-6583-treated mice. Results are expressed as mean ± SEM. n=6 mice 7 group. **, p<0.01 as determined by Student's t-test.

Based on the results shown in Figure 1, we hypothesized that A2BR activation could drive fibroblasts activation within tumor lesions. We examined the expression of FAP in melanoma sections harvested from mice treated with the selective A2BR agonist Bay60-6583 [21, 27] or vehicle. The number of FAP positive cells was significantly increased in melanoma tissues of mice treated with Bay60-6583 compared to control (Figure 2A and 2B). The percentage of FAP+ cells in tumor suspensions from Bay60-6583-treated mice versus control mice was also determined by flow cytometric analysis (Supplementary Figure S2). FAP expression was accompanied by increased expression of FGF2 (Figure 2C and 2D).

Tumor-associated fibroblasts are critical component of tumor stroma that produce and secrete various tumor-promoting factors, including CXCL12 or stromal-derived factor 1 α (SDF1α) [28–30]. Thus, we sought to examine the expression of CXCL12 in melanoma sections using a specific antibody. The results show that CXCL12 expression was higher in tissues harvested from mice treated with Bay60-6583 compared to controls (Figure 2E and 2F). The CXCL12 positive cells were FAP positive (Figure 2E and 2G), showing that fibroblasts are a major source of CXCL12 under our experimental conditions, in line with previous studies [28, 29].

### A2BR induces FGF2 and CXCL12 expression in isolated melanoma-associated fibroblasts

**Figure 3:**
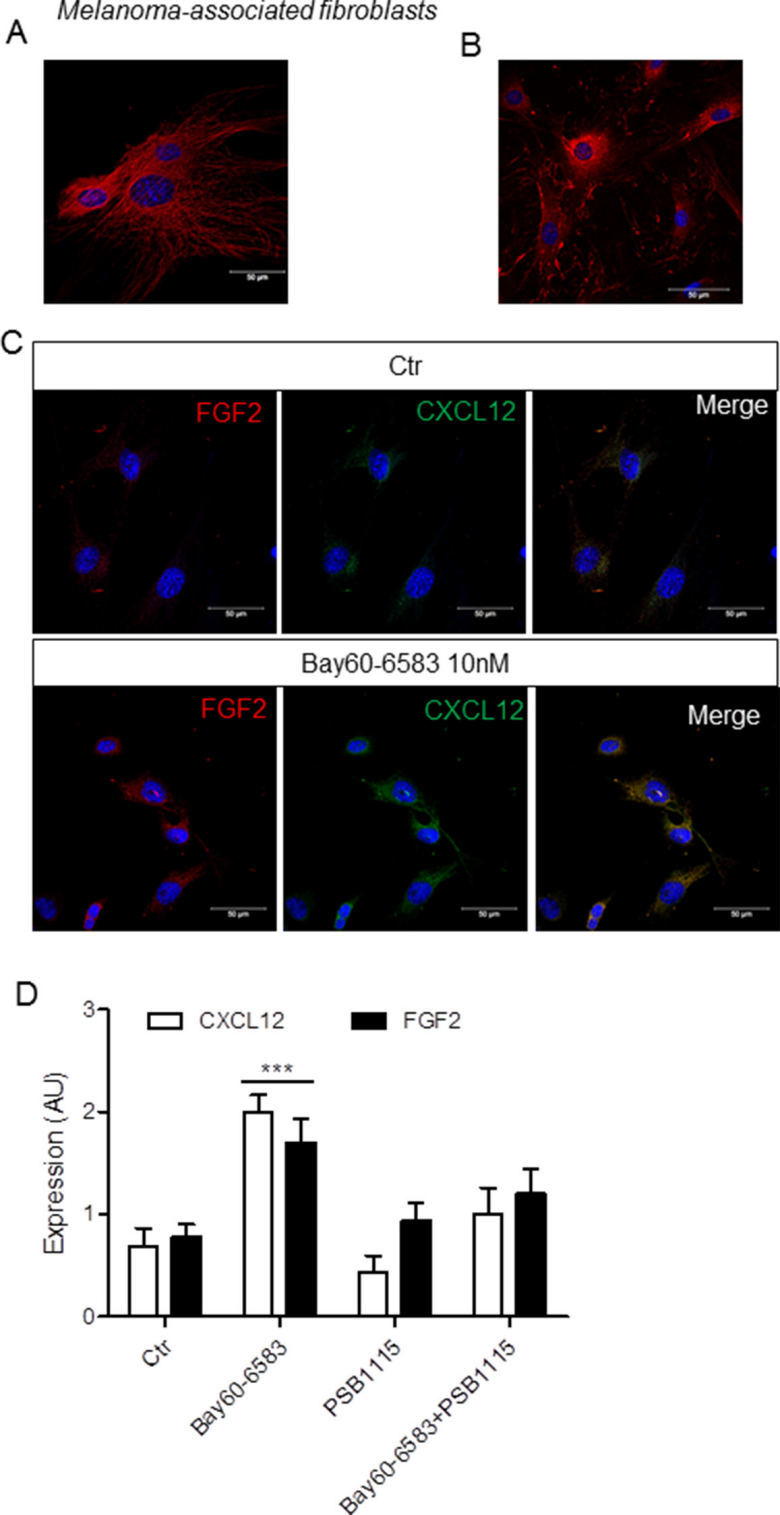
Bay60-6583 induces the expression of FGF2 and CXCL12 in isolated melanoma-associated fibroblasts **A.** and **B.** representative immunofluorescence image of melanoma-isolated fibroblasts stained with an anti-vimentin specific antibody or anti-fibronectin antibody (red), respectively, and counterstained with DAPI (blue). Scale bar, 50 μm. **C.** representative immunofluorescence images of fibroblasts isolated from melanoma tissue, grown on polylysine-coated plates and stimulated with 10 nM Bay60-6583 for 24 h or vehicle (Ctr) and then stained with an anti-FGF2 antibody (red) and with an anti-CXCL12 antibody (green) and counterstained with DAPI (blue). Scale bar, 50 μm. **D.** image analysis of CXCL12 and FGF2 in melanoma-associated fibroblasts stimulated or not with 10 nM Bay60-6583 or 100 nM PSB1115 or both for 24 hours. Results are mean (± SEM) of 4 separate fibroblast preparations, each isolated from melanoma tissue of C57B6 mice. AU, arbitrary units. ***p<0.001 as determined by ANOVA analysis.

To support a critical role of the A2BR in promoting stromal cells activation in tumors, we evaluated the response to Bay60-6583 in fibroblasts isolated from melanoma tissues of C57Bl/6 mice. Melanoma-associated fibroblasts were isolated and cultured *in vitro* as described in the Methods section. A representative image of spindle-shaped, vimentin-positive melanoma-associated fibroblasts is shown in Figure 3A. Fibronectin staining was also used to characterize isolated cells (Figure 3B). Melanoma-associated fibroblasts grown on polylysine-coated plates and treated with 10 nM Bay60-6583 for 24 hours showed increased expression of both FGF2 and CXCL12 compared to vehicle-treated cells (Ctr) (Figure 3C and 3D). These effects were abrogated by the A2B antagonist PSB1115 (100 nM, Figure 3D), suggesting that Bay60-6583 induces the expression of FGF-2 and CXCL12 in tumor-associated fibroblasts in an A2BR-dependent manner.

**Figure 4:**
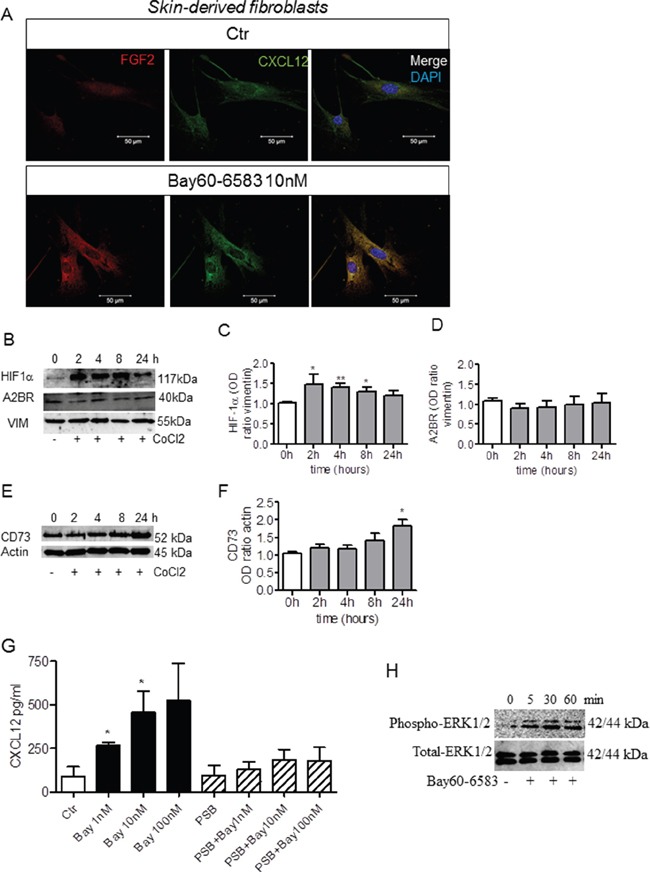
Bay60-6583 induces the expression of FGF2 and CXCL12 in skin-derived fibroblasts under hypoxic conditions in a concentration-dependent manner **A.** representative immunofluorescence images of skin-derived fibroblasts stained with an anti-FGF2 antibody (red) and with an anti-CXCL12 antibody (green) and counterstained with DAPI (blue). Cells were exposed to hypoxia (100 μM CoCl2) for 24 hours and then stimulated with 10 nM Bay60-6583 for another 24 h or vehicle (Ctr). Scale bar, 50 μm. Images are representative of separate fibroblasts preparations, each derived from skin of C57Bl/6 mice (n=5). **B.** Western blot analysis of HIF1α (upper panel) and A2B receptor (middle panel) expression in fibroblasts exposed to CoCl2 at different times (0 to 24 hours). Vimentin (lower panel) was used as loading control. **C** and **D.** The optical density of HIF-1α and A2BR, respectively, detected by Western blotting was normalized to vimentin as control and expressed as mean ±SEM. n=5 *p<0.05 and **p<0.01. **E.** Western blot analysis of CD73 expression in fibroblasts exposed to CoCl2 at different times (0 to 24 hours). β-actin was used as loading control. **F.** The optical density of CD73 is expressed as mean ± SEM. n=5 *p<0.05. **G.** ELISA analysis of CXCL12 protein levels in the supernatants of hypoxic fibroblasts stimulated with Bay60-6583 (1, 10 or 100 nM) with or without 100 nM PSB1115 (indicated as PSB) for 24 hours. Results are expressed as mean ± SEM. n=5 *p<0.05 (ANOVA). **H.** representative Western blot showing the expression of phospho-ERK1/2 and total ERK1/2 in fibroblasts control (0 min) and treated with Bay60-6583 (10 nM) at 5-30-60 min.

Normal mouse fibroblasts isolated from skin were also used to assess the response to Bay60-6583. When these cells were exposed for 24 hours to a hypoxia-inducing treatment (100 μM CoCl2) as a tumor-relevant stressor [31] and then treated with Bay60-6583 for another 24 hours, the expression of both FGF2 and CXCL12 increased (Figure 4A). The hypoxic state induced by CoCl2 resulted in hypoxia-inducible factor (HIF)-1α up-regulation (Figure 4B and 4C) [31]. The expression of A2BR was shown to be induced in hypoxia in a HIF-1-dependent manner [24, 32]. Thus, we tested whether A2BR was induced in hypoxic fibroblasts under our experimental conditions along with FGF2 and CXCL12. However, A2BR expression was not induced in hypoxic fibroblasts (Figure 4B and 4D), whilst CD73, the enzyme responsible for extracellular adenosine production, was induced (Figure 4E and 4F). The effect of Bay60-6583 on CXCL12 expression was concentration-dependent and blocked by PSB1115 (Figure 4G). We also found that stimulation of cells with Bay60-6583 (10 nM) induced a rapid phosphorylation of ERK1/2 (Figure 4H). Administration of the selective A2AR agonist CGS21680 (1 μM) did not have any effect on the expression of CXCL12 and FGF2 in isolated fibroblasts, contrary to Bay60-6583-treated cells (Supplementary Figure S3A). Moreover, in contrast to Bay60-6583, CGS21680 did not induce the expression of phospho-ERK1/2 in these cells (Supplementary Figure S3B).

### Bay60-6583-induced FGF2 enhances B16.F10 melanoma cells proliferation

**Figure 5:**
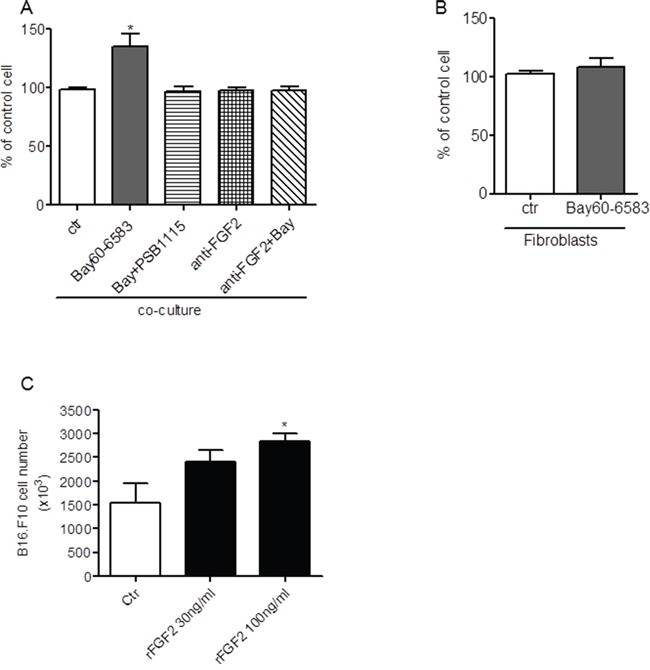
FGF2 derived from Bay60-6583 stimulated tumor-associated fibroblasts induces B16.F10 proliferation **A.** proliferation of B16.F10 melanoma cells co-cultured with tumor-associated fibroblasts (ratio 1:1) in presence of vehicle (Ctr), Bay60-6583 or anti-FGF2 antibody (anti-FGF2) or with anti-FGF2 antibody + Bay60-6583 (anti-FGF2+Bay) for 24 hours. *p<0.05 versus control (Ctr). **B.** proliferation of tumor-associated fibroblasts grown of polylysine-coated plates in presence of vehicle (Ctr) or Bay60-6583 for 72 hours. **C.** proliferation of B16.F10 melanoma cells in presence of recombinant FGF2 (30-100 ng/ml) for 24 hours. Data expressed as mean ± SEM are from two independent experiments. *p<0.05.

FGF2 is a growth factor strongly implicated in tumor cell growth [33]. To investigate whether FGF2 produced by fibroblasts upon A2BR stimulation could influence tumor cell proliferation, we performed MTT assays in B16.F10 melanoma cells co-cultured with tumor-isolated fibroblasts stimulated with 10 nM Bay60-6583 or vehicle (Ctr) for 24 hours or 48 hours. The proliferation of tumor cells co-cultured with fibroblasts increased after administration of Bay60-6583 (Figure 5A). This effect was blocked when the A2BR antagonist PSB1115 was added to the co-culture (Figure 5A). Neutralization of FGF2 with an anti-FGF2 antibody abrogated the effect of Bay60-6583 (Figure 5A). Importantly, A2BR stimulation with Bay60-6583 (for 24, 48 or 72 hours) did not affect the proliferation of isolated fibroblasts (Figure 5B), or that of B16.F10 melanoma cells in the absence of fibroblasts, as we have previously reported [17] (data not shown). Treatment with pure, recombinant FGF-b (30-100 ng/ml) recapitulated the effect of fibroblast-derived FGF-b (Figure 5C).

Taken together, these results indicate that FGF2 released from fibroblasts upon A2BR stimulation induces melanoma cells proliferation, as reported by other authors [33, 52–54].

### CXCR4 inhibition prevents the pro-angiogenic effects of Bay60-6583

**Figure 6:**
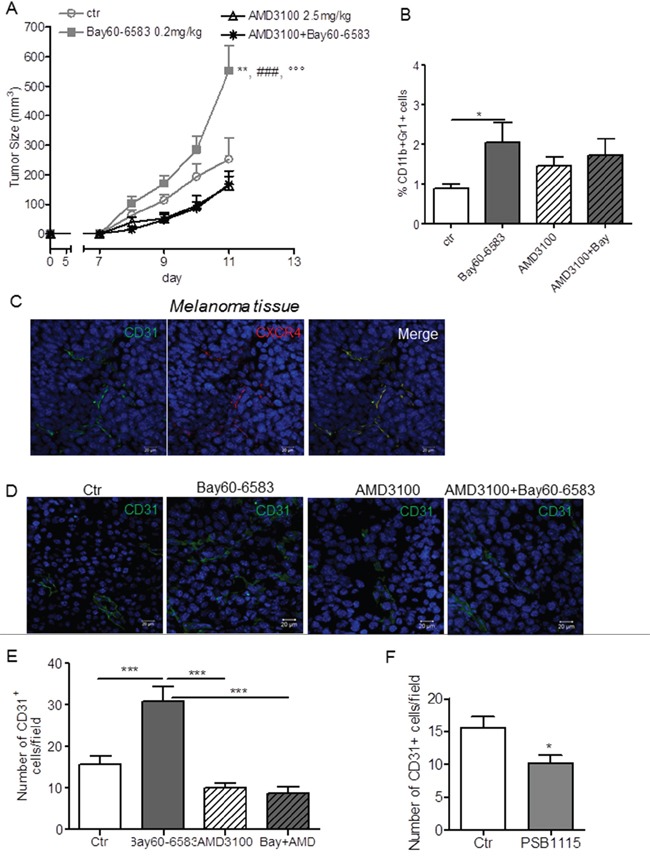
Inhibition of CXCR4 with AMD3100 prevents the pro-angiogenic effects of Bay60-6583 **A.** Tumor size (mm^3^) measured in control (Ctr) mice or mice treated with Bay60-6583, AMD3100 or AMD3100 + Bay60-6583. n=7 for control or Bay60-6583 and 10 for mice treated with AMD3100 or AMD3100 + Bay60-6583. **, p<0.01 versus Ctr, ###, p<0.001 versus AMD3100 and °°°, p<0.001 versus AMD3100 + Bay60-6583 (ANOVA). **B.** flow cytometric analysis of CD11b+Gr1+ cells measured in melanoma tissues from mice treated as above. n=7 mice/group. *p<0.05. **C.** representative immunofluorescence images of melanoma sections from mice stained with an-anti-CD31 specific antibody (FITC-conjugated) and anti-CXCR4 antibody (red) and counterstained with DAPI (blue). The merged image shows the co-localization of CD31 and CXCR4. Scale bar, 20 μm. **D.** representative immunofluorescence images of melanoma sections from control (Ctr) mice or mice treated with Bay60-6583 or AMD3100 or AMD3100 + Bay60-6583 stained with an anti-CD31 specific antibody (green) and counterstained w Scale bar, 20 μm. **E.** quantification of CD31 positive cells in tumors from mice described above. Data are from sections derived from tumors obtained from 6 different mice/group. Two sections were stained for each tumor and positive cells were counted in four to five randomly selected fields per tumor section. Results are expressed as mean ± SEM. ***p<0.001 (ANOVA). **F.** number of CD31+ cells in PSB1115-treated mice and control mice determined as described above. *p<0.05 (Student's t-test).

Next, we investigated whether A2BR-induced CXCL12 expression could contribute to the tumor promoting effects of Bay60-6583 *in vivo*. We previously described a role for A2BR in inducing tumor accumulation of MDSC, which suppress anti-tumor T-cell responses [17], and tumor angiogenesis [14]. CXCL12 and its receptor CXCR4 were shown to be important for recruitment of immunosuppressive cells such as MDSC and Tregs in the tumor microenvironment [34, 35] and for tumor angiogenesis [28]. We used a selective antagonist of the CXCL12 receptor CXCR4, AMD3100. AMD3100 was administered to melanoma-bearing mice peritumorally to directly evaluate the possible involvement of the CXCL12/CXCR4 pathway in A2BR-mediated effects within tumor environment, minimizing possible systemic effects of this molecule. Treatment with AMD3100 blocked the pro-tumor effects of Bay60-6583 and significantly reduced tumor growth in melanoma-bearing mice (Figure 6A). Tumor-infiltrating MDSCs, which express CXCR4 [34, 35] (data not shown), accumulated in mice receiving Bay60-6583 (Figure 6B). Treatment with AMD3100 did not suppress this effect (Figure 6B). No effect of AMD3100 was observed on the number of FoxP3 regulatory T cells infiltrating melanoma lesions of either control mice or Bay60-6583-treated mice (Supplementary Figure S4A). Accordingly, AMD3100 did not affect the percentage of tumor-infiltrating CD8+ or CD4+ T-cells, which were decreased in Bay60-6583-treated mice compared to controls (Supplementary Figure S4B and S4C).

CD31-positive endothelial cells also expressed CXCR4 (Figures 6C and Supplementary Figure S5) [28, 30] and A2BR stimulation increased CD31+ cells within tumor lesions (Figure 6D and 6E) [14]. Blocking CXCR4 suppressed the increase in microvessel density induced by Bay60-6583, as determined by the lower number of CD31 positive cells (Figure 6D and 6E). Notably, inhibition of A2BR in melanoma-bearing mice reduced the number of CD31+ cells compared to control (Figure 6F). Altogether, these findings suggest that A2BR-induced expression of CXCL12 in stromal cells contributes to its pro-angiogenic effect rather than immune-suppressive effects, establishing a positive cross-talk between fibroblasts and endothelial cells that sustains tumor growth.

## DISCUSSION

Stromal fibroblasts play a crucial role in tumor initiation and progression [1]. These cells participate in an intricate cross-talk with tumor cells and other stromal cells in the tumor microenvironment. One of the mechanisms of this crosstalk is the production of paracrine factors such as CXCL12 or FGF2.

Here we show that A2BR stimulation enhances the number of FAP positive cells in melanoma tissues of C57Bl6 mice. Administration of A2BR agonist Bay60-6583 enhances the expression of CXCL12 and FGF2 in melanoma-associated fibroblasts in an A2BR-dependent manner. These factors in turn contribute to promoting tumor growth. Blockade of the CXCL12 receptor CXCR4 with AMD3100 had significant anti-tumor activity and reversed the pro-angiogenic activity of Bay60-6583, but not its effects on MDSC accumulation and tumor-infiltrating Treg or CD8+T cells number.

Numerous reports show that adenosine plays an important role in modulating the functions of fibroblasts in the context of pathological processes, including wound healing and fibrosis [reviewed in Ref 36]. Due to phenotypic and functional heterogeneity of fibroblasts, different effects of A2BR stimulation have been reported. In mouse cardiac fibroblasts A2BR activation promotes IL-6 release [37], whilst it reduces collagen production and proliferation [38, 39]; in synovial fibroblasts stimulation of A2BR decreases MMP1 expression [40] and in human pulmonary fibroblasts it increases the release of IL-6 and promotes the differentiation of fibroblasts into myofibroblasts under normoxic and hypoxic conditions [24]. Here, we show for the first time, to the best of our knowledge, a role for A2BR in promoting the activation of FAP-α positive tumor stromal cells. FAP-α is expressed in activated fibroblasts during wound healing and inflammatory- and fibrosis-associated diseases [41, 42] and in quiescent mesodermal cells in multiple tissues [43]. In tumors, it has emerged as a marker of tumor-activated fibroblasts termed carcinoma-associated fibroblasts (CAFs) [1]. CAFs that selectively express FAP have been found in almost all human carcinomas [22, 44, 45], including melanoma [46]. A number of studies have shown that FAP- positive CAF promote tumor growth by inducing tumor immune evasion [29] and tumor stromagenesis and vascularization [47], making FAP a potential therapeutic target in cancer.

Our results show that the number of FAP-positive cells significantly increased in tumors of mice treated with the A2BR agonist Bay60-6583 compared to controls. This effect was associated with a higher expression of CXCL12 in FAP-positive cells. These findings were supported by *in vitro* studies in fibroblasts isolated from melanoma tissues of C57Bl6 mice exposed to Bay60-6583. The expression of CXCL12 increased in melanoma-associated fibroblasts treated with Bay60-6583. Co-administration of A2BR antagonist PSB1115 completely reversed this effect, indicating a direct role for A2BR in inducing CXCL12 production in melanoma-associated fibroblasts. Similar results were also obtained in skin fibroblasts isolated from C57Bl6 mice under hypoxic conditions, mimicking a common feature of tumor microenvironment. Based on previous reports on the up-regulation of A2BR in a HIF-dependent manner [24, 32], we hypothesized that A2BR expression was induced on our cells under hypoxic conditions. However, we were unable to detect any regulation of A2BR expression. Conversely, CD73 was up-regulated in hypoxic fibroblasts, suggesting that these cells can contribute to the production of extracellular adenosine. In the context of a tumor, high concentrations of adenosine produced by CD73-expressing fibroblasts, tumor-infiltrating immune cells and/or tumor cells [44, 45], may increase CXCL12 expression in fibroblasts themselves through A2BR. An important limitation in elucidating the specific role of the A2BR in specific cell populations is that many cells also express A2AR, which shares many similarities with A2BR. In this regard, we could not detect any effect of the A2AR agonist CGS21680 on either FGF2 or CXCL12 expression under these experimental conditions. Moreover, administration of CGS21680 did not induce ERK activation (pERK) in contrast with Bay60-6583-treated fibroblasts. These results strongly support our findings on A2B-dependent induction of FGF2 and CXCL12 expression in melanoma-derived fibroblasts, ruling out an involvement of A2AR.

A role for the A2AR in cancer-associated fibroblasts has been also identified. A study by Mediavilla-Varela et al. shows that A2AR blockade reduces the proliferation of CAF cell lines, suggesting a mechanism whereby adenosine produced by CAF enhances their proliferation in an autocrine manner via the A2AR [49]. These observations, together with the data we present here, strongly support the important role of the CD73-A2A/A2B receptors axis in modulating multiple functions of cells within tumor microenvironment, including fibroblasts.

CXCL12 regulates tumor growth, angiogenesis and progression by acting on CXCR4-expressing cells. Expression of the CXCL12/CXCR4 axis is increased in numerous cancer types [30], and it may serve as a diagnostic or prognostic biomarker in cancer patients, including those with melanoma [50, 51]. Our data indicate that A2BR stimulation can enhance the expression of CXCL12, which contributes to its pro-tumor activity. CXCL12/CXCR4 can directly influence the proliferation, migration and invasion of CXCR4-expressing tumor cells [reviewed in Ref 30]. The B16.F10 melanoma cells used in our experimental model did not express CXCL12 or CXCR4, ruling out direct effects on melanoma cells. Rather, we observed that FGF2, whose expression significantly increased in melanoma-associated fibroblasts upon A2BR stimulation, directly promotes the proliferation of B16.F10 melanoma cells. This is consistent with previous reports [52–54]. FGF2 is also an important player in tumor angiogenesis [33]. Although we did not directly address this in these studies, it is likely that this growth factor may participate to the pro-angiogenic effects of the A2BR.

AMD3100, a specific CXCR4 inhibitor, significantly suppressed tumor growth promoted by A2BR stimulation in melanoma-bearing mice. The CXCL12/CXCR4 axis promotes tumor angiogenesis through multiple mechanisms that lead to the recruitment of CXCR4 positive vascular cells into tumor lesions and/or up-regulation of pro-angiogenic factors such as VEGF or other mediators [reviewed in Ref 30]. In this study, AMD3100 completely prevented the increase in microvessel density that occurs in Bay60-6583-treated mice. Our previous work shows that A2BR stimulation induces the expression of VEGF, which facilitates tumor progression [14]. A2BR-induced CXCL12 may participate in the pro-angiogenic effect of A2BR by creating a link between stromal fibroblasts and endothelial cells.

Our results indicate that AMD3100 prevents the pro-angiogenic effects of A2BR stimulation by reducing the vessel density within tumor tissue. However, it is likely that CXCL12 also targets other cells expressing CXCR4. CXCL12 contributes to tumor immune escape, by inducing the migration of CXCR4+ regulatory cells, including MDSCs and Tregs toward CXCL12-rich tumor microenvironment [34, 35]. In this regard, some studies have demonstrated that inhibition of CXCR4 with AMD3100 improves T-cell responses in pancreatic cancer [25] or prevents immune suppression in UV-induced skin cancer [55]. In our model, AMD3100 did not suppress the accumulation of tumor MDSCs induced by Bay60-6583. No effect of AMD3100 was observed on the number of FoxP3 regulatory T-cells or CD8+T-cells infiltrated into the melanoma lesions of either control mice or Bay60-6583-treated mice. Although we cannot rule out the possibility that AMD3100 is targeting other immune-regulatory pathways that remain to be identified, the suppression of the pro-angiogenic effects induced by A2BR stimulation may be the primary mechanism of action of the antitumor effect of CXCR4 inhibition in Bay60-6583-treated mice. CXCL12 may regulate the accumulation/recruitment of immune cells through the CXCR7 receptor, which recent studies have linked to the pro-tumor effects of CXCL12 [30]. Thus, CXCR4 blockade may not be sufficient to reduce the immunosuppressive effects of A2BR. These possibilities deserve further investigation.

In summary, this study shows that A2BR activation in tumor-associated fibroblasts triggers the expression of microenvironmental tumor-promoting factors in a murine model of melanoma, including FGF2 and CXCL12. It is important to note that the pharmacological agents used in this work, with improved specificity for the A2BR, may still have off-target effects. A tissue-specific conditional knockout mouse model lacking A2BR specifically in fibroblast cells would be the ideal approach to study the effects of fibroblast-specific A2BR ablation on tumor growth. Future genetic studies will address this question. Importantly, reduced fibroblast activation within tumor stroma is responsible for a significant component of the anti-tumor effect of a selective pharmacological A2BR blocker in our experimental model. These findings contribute to provide new insights into the mechanisms through which pharmacological manipulation of A2BR regulates intercellular crosstalk in the tumor microenvironment of B16 melanoma tumors.

## MATERIALS AND METHODS

### Mice and cells

C57BL/6 mice were purchased from Charles River Laboratories (Italy). B16.F10 murine melanoma cells were purchased from American Type Culture Collection (ATCC, LCG Standards S.r.l., Milan, Italy). Animal studies were approved by Italian Health Ministry (protocol n° 12827) according to institutional animal care guidelines, Italian Law 26/2014 based on the European Community Law for Animal Care 2010/63/UE.

### *In vivo* experiments

2×10^5^ B16-F10 cells were subcutaneously (s.c.) injected in the right flank of anesthetized mice [14, 17]. At day 7 after tumor cells implantation, when palpable tumors had developed, mice were treated with the selective A2BR antagonist PSB1115 1 mg/kg [19–21] or with the selective A2BR agonist Bay60-6583 0.2 mg/kg [21, 27] (both from Sigma Aldrich, Milan, Italy) by the peritumoral (p.t.) route, to directly evaluate the effects of these molecules in tumor tissue [14, 17]. Control mice received vehicle: phosphate-buffered solution (PBS) (Euroclone, Milan, Italy) as control for PSB1115; phosphate-buffered solution containing DMSO 0.01% as control for Bay60-6583. After one week of treatment mice were sacrificed and tumor tissue was collected from each mouse for further analyses. To block CXCL12 signaling, AMD3100 2.5 mg/kg (Sigma-Aldrich, Milan, Italy), a selective antagonist of CXCR4, was administered p.t. every day starting from day 7. Tumor volume was daily monitored and calculated as previously described [14].

### Fibroblast isolation and *in vitro* experiments

To isolate tumor-associated fibroblasts melanoma samples from C57Bl6 mice were collected aseptically, washed with sterile PBS (Euroclone, Milan, Italy) and then placed in a Petri dish with RPMI1640 medium supplemented with 10% FBS, 100 U/ml penicillin, 0.1 mg/ml streptomycin (Lonza, Milan, Italy) in a tissue culture incubator, allowing fibroblasts to migrate from the tissue within 5-7 days. Fibroblasts were also isolated from skin of C57Bl6 mice as reported by Seleuanov et al., [56]. Briefly, skin samples were excised aseptically from mice and dissociated both mechanically by cutting the tissue into 1 mm pieces using a scalpel blade and enzymatically by using 1 U/ml collagenase A (Sigma-Aldrich, Milan, Italy). Digested skin fragments were placed in a Petri dish with RPMI1640 medium supplemented with 10% FBS, 100 U/ml penicillin, 0.1 mg/ml streptomycin and fibroblasts migrated out from tissue fragments within 5-7 days.

Fibroblasts isolated from either melanoma or skin tissues, grown on polylysine-coated surfaces, were treated with Bay60-6583 (1, 10 or 100 nM) for further analyses. 10 nM Bay60-6583 was selected to perform *in vitro* experiments as it achieved optimal effects in preliminary studies. Some experiments were also performed in the presence of 100 nM PSB1115. In additional experiments, fibroblasts isolated from skin tissue were exposed to 100 μM CoCl2 used as hypoxia inducer [27] at different time points (0-24 hours). Fibroblasts exposed to hypoxia were then treated with 10 nM Bay60-6583 for 24 h for further analyses. To analyze ERK1/2 activation, cells were stimulated with Bay60-6583 (10 nM) at different time points (5-30-60 min). Some experiments were also performed in presence of the A2AR agonist CGS21680 (1 μM) as described above.

### Confocal microscopy immunofluorescence

Confocal microscopy immunofluorescence analysis was performed on frozen sections of melanoma tissue or fibroblasts isolated from either melanoma or skin tissues. Frozen sections of melanoma tissue, fixed in 4% paraformaldehyde (PFA), were incubated with 20% goat serum containing 0.5% TritonX-100 for 30 minutes at room temperature to block nonspecific binding sites. Fibroblasts plated on poly-lysine-coated slides were washed twice in PBS, fixed in 4% PFA, and nonspecific binding sites were blocked using 20% goat serum or 5% bovine serum albumin (BSA) for 30 minutes at room temperature, as appropriate. Slides were stained overnight with the following primary antibodies: anti-FAPα (H-56) or anti-FGF-2 (147) (1:100; Santa Cruz Biotechnology) were detected with Alexa Fluor® 488 or 555 Goat Anti-Rabbit IgG (H+L) (1:5000) secondary antibodies (Life Technologies, Italy); anti-CXCL12 (1:100; Santa Cruz Biotechnology) (clone P-159X) was detected with the secondary antibody Alexa Fluor® 488 Goat Anti-Mouse IgG (H+L) (1:5000); anti-vimentin (E-5) (1:500; Santa Cruz Biotechnology) or anti-fibronectin (A17) (1:200; Abcam) were detected with the secondary antibody Alexa Fluor® 555 Goat Anti-Mouse IgG (H+L) (1:5000); anti-CXCR4 (clone L276F12) (1:100; BioLegend) was detected with Alexa Fluor® 555 Goat Anti-Rat IgG (H+L) (1:5000); FITC-conjugated anti-CD31 (MEC 13.3) (1:100, BioLegend) was also used. Primary antibody for A2B receptor: A2B-R (N-19) (1:50; Santa Cruz Biotechnology) was detected with the secondary antibodies DyLight™ 549-conjugated AffinePure Donkey anti-goat IgG (H+L) (Jackson ImmunoResearch Lab, UK). DAPI was used to counterstain nuclei. In all staining experiments, isotype-matched IgG and omission of the primary Ab were used as controls. Slides were observed using a Zeiss LSM 710 Laser Scanning Microscope (Carl Zeiss MicroImaging GmbH). Samples were vertically scanned from the bottom of the coverslip with a 63 × or 40 x (1.40 NA) Plan-Apochromat oil-immersion objective. Images were generated with Zeiss ZEN Confocal Software (Carl Zeiss MicroImaging GmbH).

### Western blotting

Fibroblasts were collected and suspended in RIPA buffer (Radio-Immuno Precipitation Assay Buffer). Forty micrograms of total protein were analyzed by 10% denaturing polyacrylamide gels and then transferred electrophoretically to PVDF membranes (Immobilon-NC, Millipore, Italy). Anti-A2BR (H-40) (Santa Cruz Biotechnology), anti HIF1α (A300-286A) (Bethyl Laboratories, Tema Ricerca, Italy) or anti CD73 (5NT5E, C-terminal) (Sigma-Aldrich, Milan, Italy) or phospho-p44/42 MAPK (Erk1/2) (Thr202/Tyr204) (D13.14.4E) or p44/42 MAPK (Erk1/2) (137F5) (Cell Signaling Technology) primary antibodies were used. Immunoreactive protein bands were visualized by enhanced chemiluminescence reagents (Amersham Pharmacia Biotech, Buckinghamshire, UK) and analyzed to Las4000 (GE Healthcare Life Sciences).

### Proliferation assay

Tumor-derived fibroblasts, B16-F10 cells or co-cultures of tumor-derived fibroblasts and B16-F10 cells (1:1 ratio) were seeded onto 96 well plates and treated with 10 nM Bay60-6583, 100 nM PSB1115 or both, and cell proliferation assays were performed at 24-48-72 hours. Some experiments with fibroblasts co-cultured with melanoma cells were performed in presence of an anti-FGF2 antibody (100 ng/ml) alone or in presence of 10 nM Bay60-6583. Recombinant FGF-β (Lonza, Italy) was used at the concentrations of 30-100 ng/ml.

### ELISA

Mouse CXCL12/SDF-1 alpha ELISA kit (R&D Systems, Abingdon, UK) was used to measure the levels of CXCL12 in the supernatant of isolated fibroblasts stimulated with 1, 10 or 100 nM Bay60-6583 for 24 h with or without 100 nM PSB1115.

### Flow cytometry

Melanoma tissues were digested with 1 U/ml collagenase (Sigma Aldrich, Italy), passed through 70-μm cell strainers and red blood cells (RBC) were lysed to prepare single cell suspensions. Cell samples were blocked with anti-mouse CD16/CD32 (eBioscience, San Diego, CA, USA). Antibodies against CD11c-FITC, CD11b-PeCy5.5, Gr1-PE or Gr1-allophycocyanin, CD3-PeCy5.5; CD8-allophycocyanin or CD8-PE; CD4-allophycocyanin; NK1.1-PE were obtained from eBioscience and BioLegend. Expression of FAP in melanoma tissue was also analyzed by flow cytometry by using an anti-FAP-FITC and expressed as percentage of positive cells. Data were acquired with a FACSCalibur flow cytometer (BD Biosciences).

### Statistics

Results of confocal microscopy immunofluorescence are coming from sections derived from tumors obtained from at least 5-6 different mice per condition, as reported in the figure legends. Two sections were stained for each tumor and positive cells were counted in four-to-five randomly selected fields per tumor section. The results of immunofluorescence in fibroblasts are expressed as mean of 4 separate fibroblast preparations, each isolated from C57Bl6 mice (±S.E.M.). The optical density of the protein bands detected by Western blotting was performed with ImageQuant^TL^ (Ge Healthcare). Results are expressed as mean ± SEM. Data were analyzed with GraphPad Prism 6 (GraphPad Software). Two-tailed Student's *t* test or ANOVA were used as appropriate. P values < 0.05 were considered significant and reported in the figure legends.

## SUPPLEMENTARY MATERIALS FIGURES


