# Supplementary material for: Activation of the A2B adenosine receptor in B16 melanomas induces CXCL12 expression in FAP-positive tumor stromal cells, enhancing tumor progression
Source: Oncotarget. 2016 Aug 31;7(39):64274–88. doi: 10.18632/oncotarget.11729 (PMC5325441; doi:10.18632/oncotarget.11729)
Supplement: Supplementary file 1 [file oncotarget-07-64274-s001.pdf]

Activation of the A2B adenosine receptor in B16 melanomas induces CXCL12 expression in FAP-positive tumor stromal cells, enhancing tumor progression

SUPPLEMENTARY FIGURES

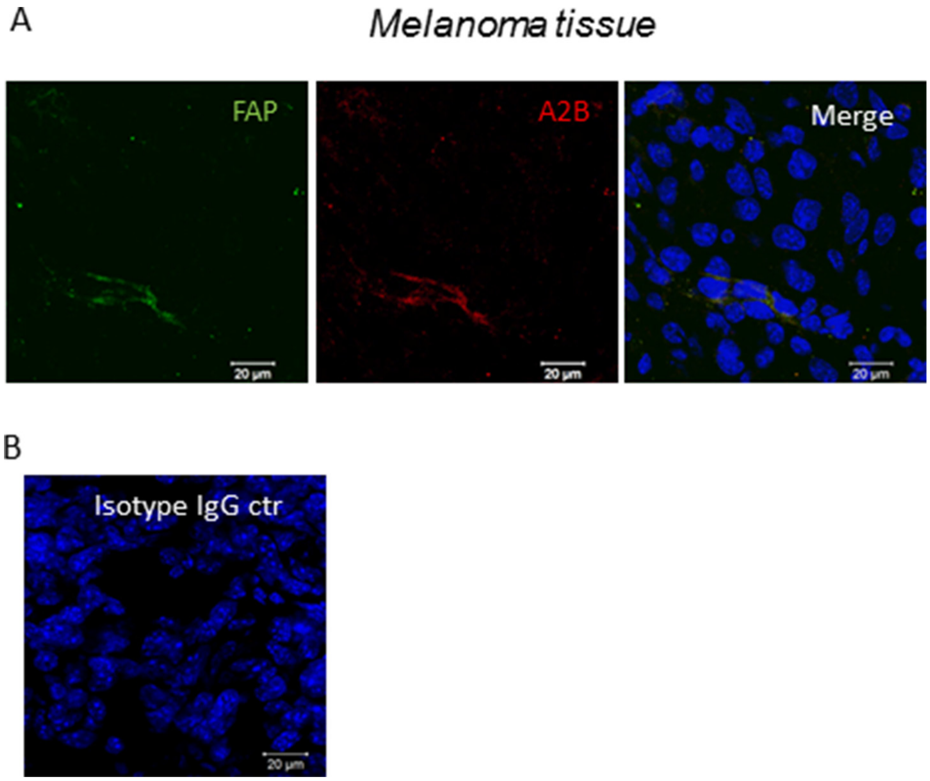

Supplementary Figure S1: FAP positive cells in melanoma tissues express A2BR. A. representative immunofluorescence images of melanoma sections from C57Bl/6 mice stained with anti-FAPα antibody (green) and with an anti-A2B receptor specific antibody (red) and counterstained with DAPI (blue). The merged image shows the co-localization of A2B and FAP. Scale bar, 20 µm. **B.** immunofluorescence image of melanoma section stained with isotype IgGs control. Scale bar, 20 µm.

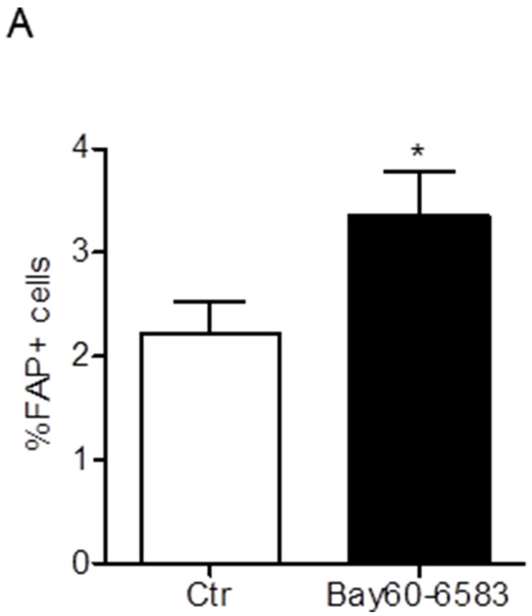

Supplementary Figure S2: FAP expression enhances in melanoma tissue of Bay60-6583-treated mice. A. percentage of FAP+ cells analyzed by flow cytometry in melanoma tissues harvested from control mice and mice treated with Bay60-6583 (0.2 mg/kg). Data are mean ± SEM. n=6 mice / group. *$p<0.05$ as determined by Student's t-test.

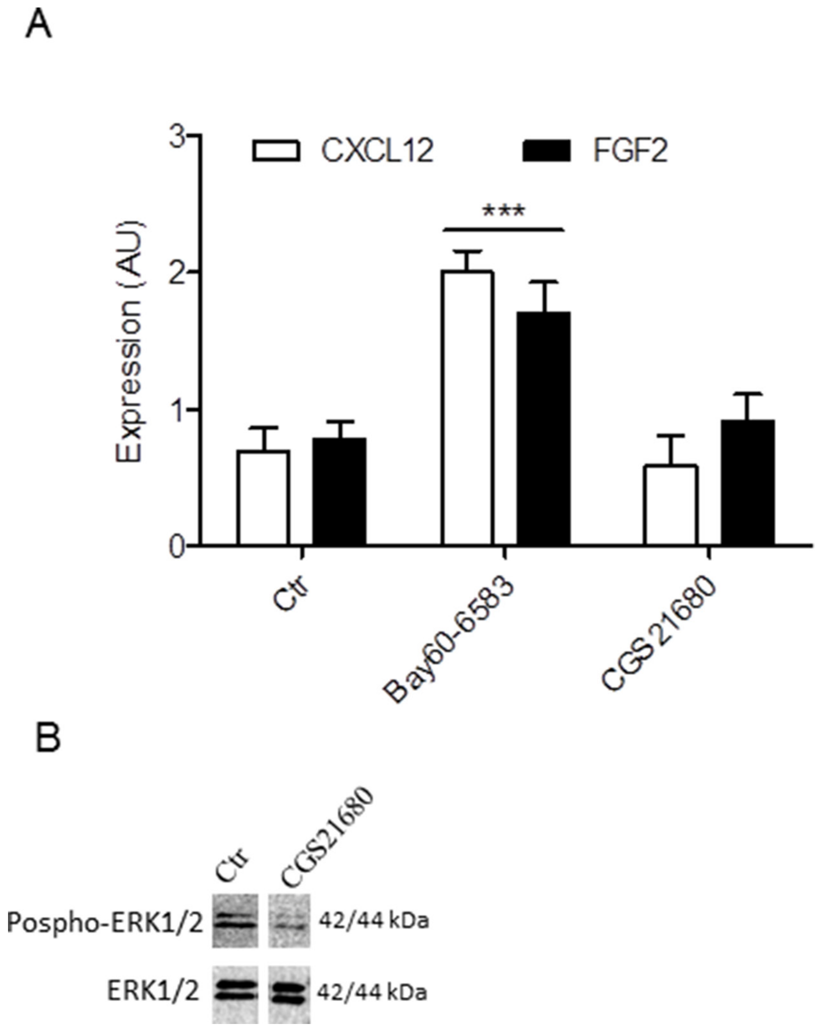

Supplementary Figure S3: CGS21680 treatment of isolated fibroblasts. A. melanoma-associated fibroblasts were treated with the selective A2AR agonist CGS21680 (1 µM) for 24 h. Expression of CXCL12 and FGF2 was unaltered in cells treated with CGS21680 in contrast to those observed in cells treated with Bay60-6583. Fibroblasts were isolated from melanoma tissue of C57Bl6 mice (n=3). Data are mean ± SEM. ***p<0.001 (ANOVA). **B.** representative Western blot showing phospho-ERK1/2 and total ERK1/2 expression in control fibroblasts and fibroblasts treated with CGS21680.

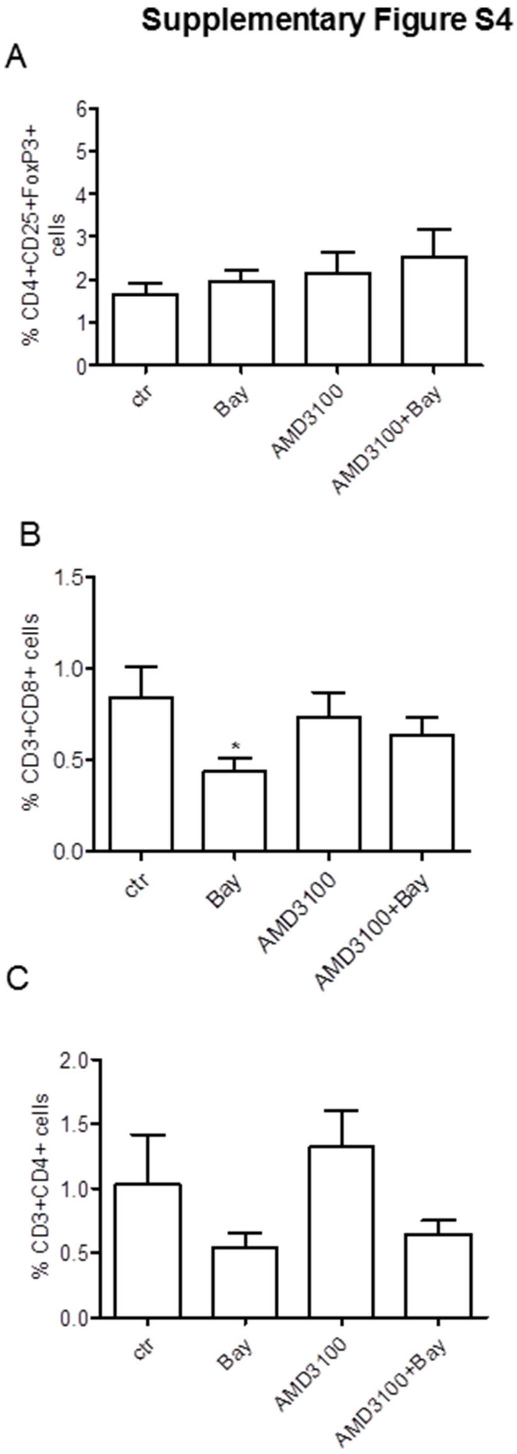

Supplementary Figure S4: Tumor-infiltrating T cells in mice treated with Bay60-6583 or AMD3100 or AMD3100 + Bay60-6583. A. flow cytometric analysis of CD4+CD25+FoxP3+T cells measured in melanoma tissues from control (Ctr) mice or mice treated with Bay60-6583 (Bay) or AMD3100 or AMD3100 + Bay60-6583 (AMD3100+Bay). **B.** flow cytometric analysis of CD3+CD8+T cells measured in melanoma tissues from mice treated as above. **C.** flow cytometric analysis of CD3+CD4+T cells measured in melanoma tissues from mice treated as above. Results are expressed as mean ± SEM. n=7 for mice control or treated with AMD3100 and 8 for mice treated with Bay60-6583 or AMD3100 + Bay60-6583. *, $p<0.05$ versus Ctr.

CD31-FITC + normal rat IgG control

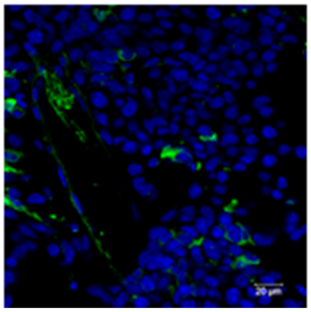

Supplementary Figure S5: Representative immunofluorescence image of melanoma section stained with an anti-CD31-FITC-conjugated antibody and normal rat IgG control. Scale bar, 20 μm.
